# The Role and Underlying Mechanisms of Exercise in Heart Failure

**DOI:** 10.31083/j.rcm2508285

**Published:** 2024-08-12

**Authors:** Chong-Yi Zhang, Ku-Lin Li, Xiao-Xi Zhao, Zhen-Ye Zhang, An-Wen Yin, Ru-Xing Wang

**Affiliations:** ^1^Department of Cardiology, The Affiliated Wuxi People's Hospital of Nanjing Medical University, 214023 Wuxi, Jiangsu, China

**Keywords:** exercise, heart failure, cardiac function, mechanism

## Abstract

Heart failure is a prevalent and life-threatening syndrome characterized by structural and/or functional abnormalities of the heart. As a global burden with high rates of morbidity and mortality, there is growing recognition of the beneficial effects of exercise on physical fitness and cardiovascular health. A substantial body of evidence supports the notion that exercise can play a protective role in the development and progression of heart failure and improve cardiac function through various mechanisms, such as attenuating cardiac fibrosis, reducing inflammation, and regulating mitochondrial metabolism. Further investigation into the role and underlying mechanisms of exercise in heart failure may uncover novel therapeutic targets for the prevention and treatment of heart failure.

## 1. Introduction

Heart failure (HF) is a multifaceted clinical syndrome that represents the final outcome of various cardiovascular diseases (CVD). Epidemiological data reveals that HF affects over 64 million individuals worldwide, resulting in significant morbidity and mortality, diminished quality of life, and substantial costs [1]. Despite advancements in guideline-directed diagnosis and treatment for HF, it remains a pressing public health issue and a global burden. Recent research has demonstrated that HF is not solely limited to the elderly population, as an increasing number of younger patients are being admitted to hospitals due to HF at an alarming rate [2]. Consequently, it is of utmost importance to investigate the specific mechanisms underlying HF and explore novel therapeutic approaches.

**Fig. 1.**
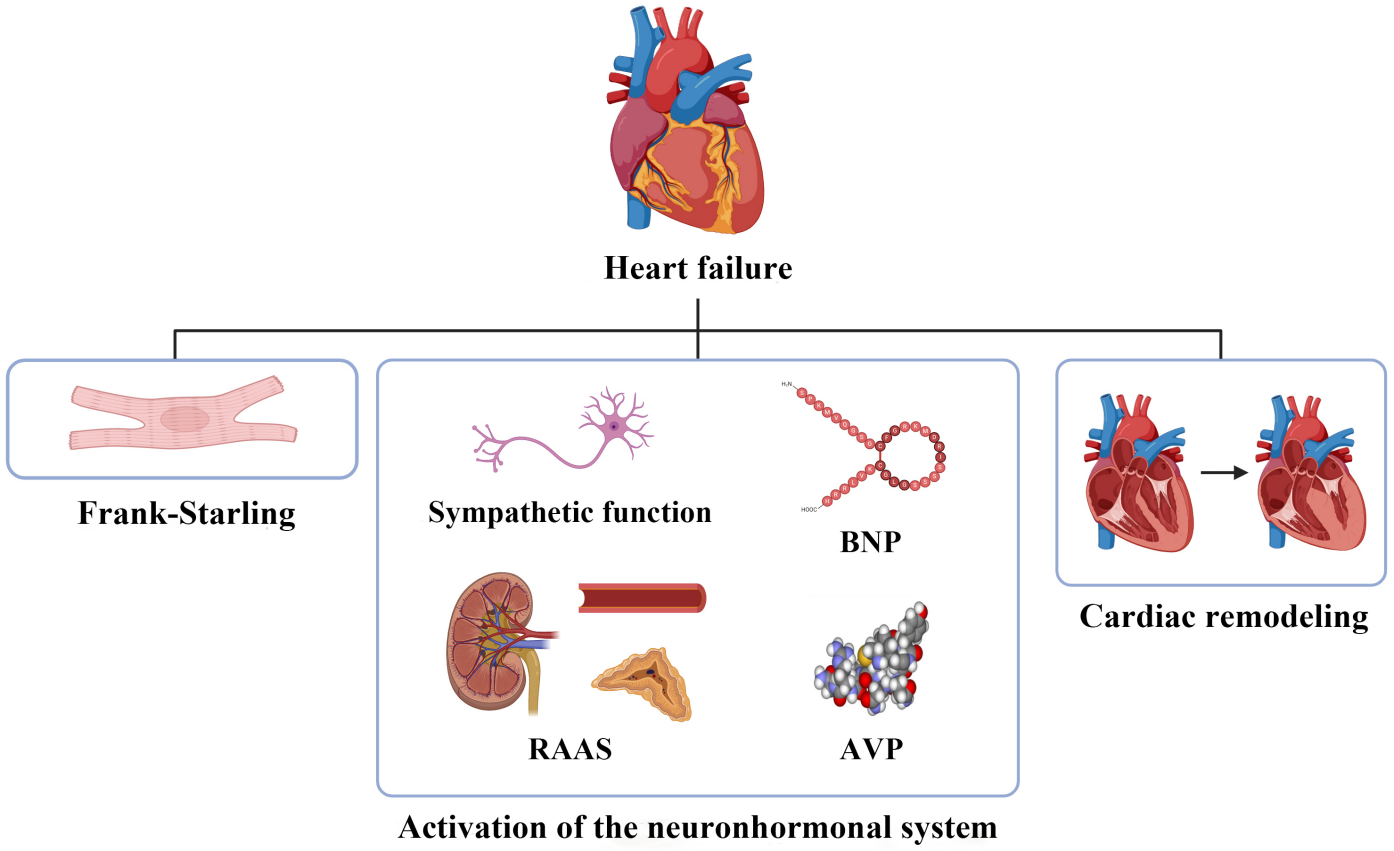
**The pathophysiology of heart failure**. BNP, brain natriuretic peptide; RAAS, renin–angiotensin–aldosterone system; AVP, arginine vasopressin.

HF is characterized by structural or functional disorders affecting the filling and/or ejection of blood from the ventricles. Based on the left ventricular ejection fraction (EF), HF can be classified into three phenotypes: HF with preserved EF (HFpEF), mildly reduced EF (HFmrEF), and reduced EF (HFrEF) [3]. Currently, the primary mechanisms involved in HF include the Frank-Starling mechanism, activation of the neurohormonal system, and cardiac remodeling [4]. The activation in the neurohormonal system includes the sympathetic nervous system, the renin–angiotensin–aldosterone system and the secretion of humoral factors such as natriuretic peptides and arginine vasopressin [5, 6] (Fig. 1). However, the underlying molecular mechanisms warrant further investigation.

Exercise is widely recognized as a healthy lifestyle choice and a non-drug therapeutic and preventive strategy for various diseases. A growing evidence indicates that exercise can maintain and restore homeostasis at multiple levels, including the organismal, tissue, cellular, and molecular levels. This, in turn, triggers positive physiological adaptations that protect against a range of pathological stimuli [7].

Regarding cardiovascular health, exercise consistently demonstrates beneficial effects across multiple aspects. These include reductions in resting heart rate and blood pressure, improvements in lipid profiles, enhanced autonomic tone, weight loss, and metabolic changes leading to improved glucose tolerance [8]. Furthermore, exercise plays a crucial role in reducing cardiovascular risk factors and the incidence of cardiovascular events. A population-based cohort study shows that physical activity levels are associated with reduced mortality risk in individuals with or without CVD. Notably, individuals with CVD may derive even greater benefits from physical activity than healthy subjects without CVD [9]. Regular exercise, guided by optimal prescription, can enhance exercise capacity and cardiorespiratory fitness, reduce hospitalization and mortality rates, and improve the quality of life in patients with hypertension, coronary heart disease, cardiomyopathy, and HF [10]. The cardioprotective effects of exercise are thought to be mediated by enhancing antioxidant capacity, promoting physiological cardiac hypertrophy, and inducing vascular and cardiac metabolic adaptations, among other mechanisms [11].

This review primarily focuses on the beneficial effects of exercise in protecting against the development and progression of HF, as well as the underlying mechanisms involved.

## 2. Role of Exercise in Heart Failure

Emerging evidence strongly supports the crucial role of exercise in both primary and secondary prevention of HF. Exercise has been shown to reduce the incidence of HF in individuals before the onset of HF, as well as delay the progression of HF. A longitudinal study conducted by Kraigher-Krainer *et al*. [12] examined 1142 elderly participants from the Framingham Study over a 10-year follow-up period. The study revealed a significant association between lower physical activity levels and a higher incidence of HF. Prospective observational studies have consistently demonstrated that higher levels of total physical activity, leisure-time activity, and occupational activity are associated with a statistically significant decreased risk of developing HF [13]. A UK Biobank prospective cohort study of 94,739 participants indicated that 150 to 300 minutes/week of moderate-intensity exercise and 75 to 150 minutes/week of high-intensity exercise can reduce the risk of HF by 63% and 66% respectively [14]. These studies collectively underscore the importance of exercise in the primary prevention of HF.

Furthermore, exercise has been found to protect against the progression of HF. The Heart Failure: A Controlled Trial Investigating Outcomes of Exercise Training (HF-ACTION) trial assigned 2331 medically stable outpatients with HFrEF to either an exercise training group or a usual care group. The results showed that exercise training was associated with significant reductions in all-cause mortality, cardiovascular mortality, and HF hospitalization [15]. In a prospective randomized controlled trial, it was demonstrated that one year of dedicated exercise training significantly reduced left ventricular chamber and myocardial stiffness constants and preserved myocardial compliance in patients with stage B HFpEF [16].

The benefits of exercise are also observed in acute HF patients. A study involving a diverse population of older patients hospitalized for acute decompensated HF (ADHF) demonstrated that an early, transitional, progressive, multi-domain physical rehabilitation intervention based on individualized exercise prescription led to improved physical function and quality of life [17]. At 6 months, there was a non-significant 10% lower rate of all-cause hospitalizations in the intervention group. Subgroup analyses by HF phenotypes revealed that the physical rehabilitation intervention may reduce both mortality and rehospitalizations in patients with HFpEF, while the intervention appeared to have relatively less benefit in patients with HFrEF, as reported by Mentz *et al*. [18]. Moreover, Pandey *et al*. [19] demonstrated that elderly ADHF patients with worse baseline frailty status experienced more significant improvement in physical function in response to the multi-domain physical rehabilitation intervention. These findings highlight the greater benefits that early exercise rehabilitation intervention can provide to ADHF patients.

Not only clinical research but also animal experiments support the benefits of exercise in HF. Mice injected with isoproterenol (ISO) exhibited significant impairment in cardiac contractile function, while exercise effectively inhibited ISO-induced HF, leading to increased fraction shortening, EF, cardiac output, and stroke volume [20]. In a rat model of myocardial infarction (MI), exercise training improved post-MI cardiac function and mitigated MI-induced ventricular pathological remodeling [21]. Exercise also attenuated diastolic and contraction function disorders and alleviated cardiac dysfunction in db/db mice [22].

## 3. Potential Mechanisms of Exercise in Heart Failure

**Fig. 2.**
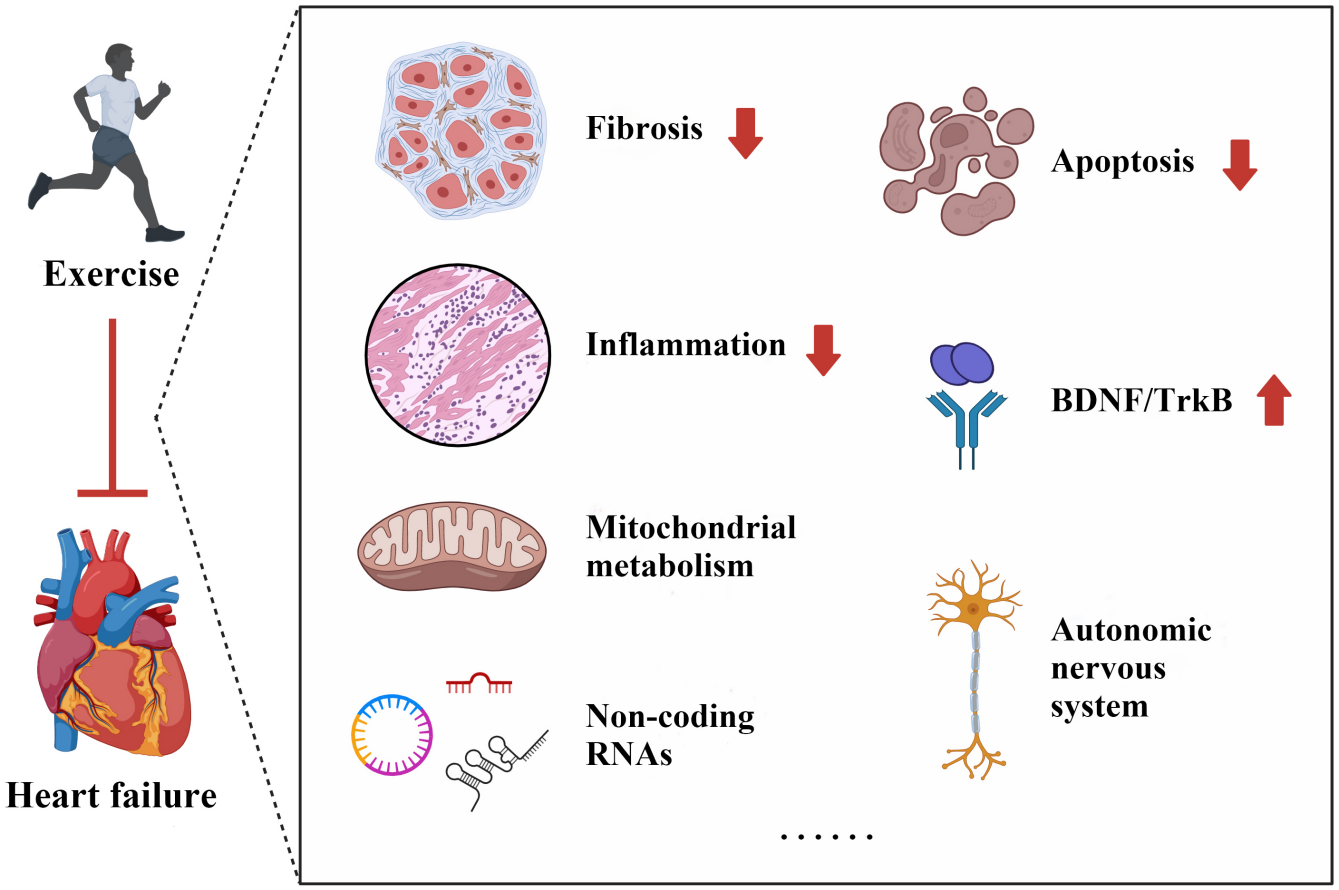
**Potential mechanisms of exercise in protecting against heart failure**. BDNF, brain-derived neurotrophic factor; TrkB, tropomyosin-related kinase B.

Exercise plays a crucial role in maintaining cardiovascular health and providing protection against HF. This beneficial effect is attributed to various underlying mechanisms, including the alleviation of cardiac fibrosis, reduction of inflammation, regulation of mitochondrial metabolism, and mediation of non-coding RNAs (ncRNAs), among others (Fig. 2).

### 3.1 Alleviating Cardiac Fibrosis

Cardiac fibrosis refers to the excessive deposition of extracellular matrix components and the replacement of normal myocardium with collagen-based fibrotic tissue, leading to impaired systolic and diastolic function of the heart [23]. It is a key pathological process in cardiac remodeling and plays a significant role in the development and progression of HF.

Transforming growth factor-beta (TGF-β) is a growth factor that can activate fibroblasts and induce profibrotic responses through its canonical pathway. Emerging evidence suggests that exercise can improve cardiac function and prevent HF by targeting TGF-β. Ma *et al*. [24] conducted a study using a mouse model of MI and subjected the mice to different types of exercise training for five weeks. Their findings revealed that both aerobic and resistance exercise training up-regulated the expression of fibroblast growth factor 21 protein, inactivated the TGF-β1-mothers against DPP homolog 2/3 (Smad2/3)-matrix metalloproteinase 2/9 (MMP2/9) signaling pathway, reduced collagen production, alleviated cardiac fibrosis, and ultimately improved cardiac function in MI mice. Chen *et al*. [25] demonstrated that exercise could down-regulate the expression of phosphodiesterase 10A (PDE10A). PDE10A deficiency or inhibition further reduced TGF-β-stimulated activation, proliferation, migration of cardiac fibroblasts, as well as extracellular matrix synthesis. This attenuation of cardiac fibrosis and dysfunction was observed in models induced by Angiotensin II (Ang II) or transverse aortic constriction (TAC).

Adenosine monophosphate-activated protein kinase (AMPK) is an endogenous protective factor for the heart that can be activated by exercise. Multiple studies have associated the beneficial effects of exercise with AMPK activation and subsequent attenuation of cardiac fibrosis. Ma *et al*. [26] reported that swimming exercise mitigated cardiac fibrosis and prevented the development of ISO-induced HF by inhibiting the nicotinamide adenine dinucleotide phosphate (NADPH) oxidase-reactive oxygen species (ROS) pathway through AMPK activation. Exercise training also activated AMPK and inhibited the increase in CCAAT enhancer-binding protein β (C/EBPβ) and POU domain, class 2, transcription factor 1 (POU2F1) induced by Ang II, thereby attenuating cardiac fibrosis and improving cardiac diastolic function [27].

In summary, exercise training has been shown to exert beneficial effects in protecting against HF by alleviating cardiac fibrosis. This is achieved through various mechanisms such as targeting TGF-β, activating AMPK, and modulating associated signaling pathways.

### 3.2 Reducing Inflammation

Inflammation has been widely recognized as an independent risk factor for HF, with immune activation and excessive production of inflammatory cytokines contributing to its pathogenesis [28]. Recent studies have highlighted the potential of exercise in delaying the development and progression of HF through its anti-inflammatory effects.

Feng *et al*. [20] demonstrated that exercise can increase the population of myeloid-derived suppressor cells by stimulating the secretion of interleukin-10 (IL-10) from macrophages. This effect was mediated through the IL-10/signal transducer and activator of transcription 3 (STAT3)/S100 calcium-binding protein A9 (S100A9) signaling pathway, providing protection against the progression of HF. Alemasi *et al*. [29], using a cytokine antibody array, observed that running exercise inhibited the expression of inflammatory cytokines following acute β-adrenergic receptor overactivation, thereby attenuating cardiac diastolic dysfunction and preventing the development of HF. Zhang *et al*. [30] identified that exercise training could alleviate macrophage infiltration and cardiac inflammation induced by ISO, resulting in improved cardiac function. This effect was achieved by inhibiting the ROS-NOD-like receptor family pyrin domain-containing 3 (NLRP3) inflammasome signaling pathway through the activation of AMPK. Additionally, another study demonstrated that early moderate-intensity aerobic exercise reduced the expression of IkappaBalpha (IκBα), nuclear factor-kappa B (NF-κB), cyclooxygenase-2 (COX-2), and IL-8, leading to decreased inflammatory responses, improved myocardial remodeling, and enhanced cardiac function in doxorubicin-induced HF [31]. Therefore, one of the novel mechanisms by which exercise protects against HF is by alleviating inflammation.

Collectively, these findings highlight the potential of exercise to reduce inflammation, which plays a significant role in protecting against the development and progression of HF.

### 3.3 Regulating Mitochondrial Metabolism

Mitochondria, which are highly concentrated in cardiomyocytes, play a vital role in cell metabolism and are crucial for meeting the energy demands of cardiac contractile function [32]. The benefits of exercise in HF are closely linked to mitochondrial metabolism and the associated intracellular signaling processes.

Campos *et al*. [33] demonstrated that exercise can improve mitochondrial morphology in failing hearts by restoring the balance between mitochondrial fission and fusion and reducing the accumulation of fragmented mitochondria. Following MI, mitochondria exhibit moderate swelling, vacuolar degeneration, and cristae disruption. However, post-infarction exercise has been shown to improve mitochondrial membrane integrity, morphology, and biogenesis [21].

In addition to improving mitochondrial morphology, exercise protects against HF by regulating mitochondrial function. Exercise has been found to restore autophagic flux, enhance tolerance to mitochondrial permeability transition, and reduce mitochondrial release of ROS in HF [33]. α-ketoglutaric acid (AKG), an important intermediate in the tricarboxylic acid cycle, is elevated after acute and resistance exercise. An *et al*. [34] discovered that AKG protects mice with TAC-induced myocardial hypertrophy and left ventricular systolic dysfunction by promoting myocardial mitophagy and reducing ROS production. Jia *et al*. [21] demonstrated that post-infarction exercise significantly attenuates MI-induced mitochondrial damage and oxidative stress by enhancing the total antioxidant capacity, activating the sirtuin 1 (SIRT1)/peroxisome proliferator-activated receptor gamma coactivator-1alpha (PGC-1α)/phosphatidylinositol 3-kinase (PI3K)/protein kinase B (Akt) signaling pathway, and alleviating cardiac dysfunction. Deloux *et al*. [35] reported that voluntary exercise improves cardiac function and prevents pathological cardiac remodeling in non-ischemic dilated HF by increasing mitochondrial aconitase activity and enhancing mitochondrial metabolism.

In summary, exercise exerts its beneficial effects in HF and cardiac function by improving mitochondrial morphology, regulating mitochondrial function, and activating intracellular signaling pathways. These mechanisms include promoting mitochondrial fission-fusion balance, enhancing mitochondrial biogenesis, restoring autophagy, reducing ROS release, and increasing mitochondrial metabolism.

### 3.4 Mediating ncRNAs

ncRNAs are a class of RNAs that are transcribed from DNA but do not encode protein. Owing to the growing technology of genomics, ncRNAs have increasingly gained attention in CVD. Extensive studies have focused on the dynamic regulation of ncRNAs by exercise, revealing their potential to protect the heart against HF.

MicroRNAs (miRNAs) are novel ncRNAs that play functional roles in exercise and their ability to mitigate cardiac pathologies. miR-222 participates in exercise-induced growth of cardiomyocytes and overall heart size. Liu *et al*. [36] investigated the effect of exercise on circulating miR-222 levels in patients with chronic stable HF. They found that miR-222 levels increased significantly after exercise, similar to what has been observed in healthy young athletes. Furthermore, using murine models of exercise (ramp swimming and voluntary wheel running), they observed robust upregulation of miR-222, which conferred resistance to pathological cardiac remodeling and dysfunction after ischemic injury. Another study by Zhou *et al*. [37] confirmed the role of miR-222 and its protective effects. They demonstrated that exercise-induced elevation of miR-222, which targets homeodomain-interacting protein kinase 2 (HIPK2), reduced infarct size and protected against post-MI cardiac dysfunction. Conversely, cardiac dysfunction following MI was aggravated in miR-222 knockout rats.

In addition to miR-222, several other miRNAs have been implicated in exercise-related cardioprotection. Shi *et al*. [38] reported that miR-17-3p was elevated in murine exercise models and that mice injected with a miR-17-3p agomir were protected against maladaptive remodeling and HF following cardiac ischemia/reperfusion (I/R) injury, by targeting metallopeptidase inhibitor 3 (TIMP3) and the phosphatase and tensin homolog deleted on chromosome 10 (PTEN)-Akt pathway. Bei *et al*. [39] observed that swimming exercise significantly induced miR-486 expression. They further demonstrated that the beneficial effect of miR-486 in attenuating cardiac remodeling and dysfunction after I/R injury was evident through the use of adeno-associated virus serotype 9 (AAV9) expressing miR-486 with a cardiac muscle cell-specific promoter (cTnT). Lew *et al*. [40] discovered that early exercise intervention upregulated miR-126, miR-499, miR-15b, and miR-133 in db/db mice, leading to improved cardiac structural remodeling and impeding the onset and progression of HF in the diabetic heart. Additionally, aerobic exercise training was shown to restore cardiac miR-1 and miR-29c to nonpathological levels, counteracting cardiac dysfunction in obese Zucker rats [41].

Long non-coding RNAs (lncRNAs) have been well-documented in cardiac development, pathological hypertrophy and HF. Exercise can regulate cardiac lncRNA, long noncoding exercise-associated cardiac transcript 1 (lncExACT1), which induces various exercise-related cardiac phenotypes, including physiological hypertrophy, improved cardiac function, and protection against HF [42]. Gao *et al*. [43] identified an exercise-induced lncRNA CPhar and found that overexpression of CPhar prevented myocardial I/R injury and cardiac dysfunction.

Circular RNAs (circRNAs) have also emerged as pivotal players in HF. Exercise can significantly increase the circUtrn level, while circUtrn suppression inhibits the protective effects of exercise on I/R-induced cardiac dysfunction [44]. In addition, exercise preconditioning up-regulates circ-Ddx60 and manifests its inhibitory effect on pathological cardiac hypertrophy as well as its protection on HF induced by TAC [45].

These findings highlight the involvement of various ncRNAs on the beneficial effects of exercise on the heart. These ncRNAs, through their regulatory functions, contribute to protection against cardiac remodeling, dysfunction, and HF in response to exercise.

### 3.5 Other Potential Mechanisms

There are still several potential mechanisms through which exercise protects against HF, and further research is needed to fully elucidate these complex mechanisms.

Excessive apoptosis has been implicated in left ventricular regeneration and the development of HF. Emerging studies suggest that exercise may reduce apoptosis, thereby exerting cardioprotective effects. For instance, exercise-induced myonectin has been shown to reduce cardiomyocyte apoptosis through the sphingosine-1-phosphate (S1P)-dependent activation of the cyclic adenosine monophosphate (cAMP)/Akt pathway, contributing to the improvement of cardiac dysfunction following I/R injury [46]. In mice with type 2 diabetes, aerobic exercise has been found to inhibit P2X purinoceptor 7 (P2X7) purinergic receptors, resulting in reduced Caspase-3 levels, a decreased Bcl2 associated X protein (Bax)/B-cell lymphoma/leukemia 2 (Bcl2) ratio, and suppressed myocardial apoptosis, leading to improved cardiac remodeling and relieved cardiac dysfunction [22].

Brain-derived neurotrophic factor (BDNF) is a critical neurotrophin in maintaining cardiac homeostasis by activating its specific receptor, tropomyosin-related kinase B (TrkB) [47]. It is noteworthy that BDNF/TrkB signaling can arrest chronic HF progression [48], while exercise can promote BDNF expression and signaling [49]. In response to exercise, BDNF/TrkB signaling increases the expression of PGC-1α and other cardiac metabolic transcription regulators, thus protecting against the progression of HF [50]. Zhang *et al*. [51] demonstrated that exercise training in MI-induced HF mice upregulated BDNF, p-TrkB, p-AMPK and PGC-1α levels, and prevented cardiac dysfunction. Lee *et al*. [52] found that the exercise-induced improvement of cardiac function is mediated by the BDNF-TrkB axis and the downstream effectors ${\mathrm{Ca}}^{2+}$/calmodulin-dependent protein kinase II (CaMKII) and Akt.

In HF, the imbalance of the autonomic nervous system is reflected by increased sympathetic nervous system activity [53]. Accumulating studies indicate that exercise training can decrease sympathetic activity, as assessed by heart rate recovery and variability, and improve autonomic function in HF patients [54]. Mechanically, exercise training restores activated sympathetic drive and attenuates cardiac dysfunction in chronic HF by increasing nitric oxide (NO) bioavailability [55]. Wafi *et al*. [56] found that exercise can upregulate both the antioxidant transcription factor Nrf2 and the antioxidant enzyme NAD(P)H quinone dehydrogenase 1 (NQO1), which reduces sympathetic function in mice with HF. Furthermore, exercise can release C-terminal coiled-coil domain-containing protein 80 (CCDC80tide), a peptide, into the circulation through extracellular vesicles. This peptide restricts pro-remodeling Janus kinase 2 (JAK2)/STAT3 signaling activity and protects against Ang II-induced cardiomyocyte hypertrophy [57]. Exercise training has also been shown to improve cardiac dysfunction and reverse HFpEF phenotypes by modifying N6-methyladenosine and downregulating fat mass and obesity-related protein levels in a mouse model [58]. Additionally, endurance exercise training can reduce cardiac mRNA m6A methylation levels, downregulate methyltransferase like 14 (METTL14) and regulate PH domain and leucine rich repeat protein phosphatase 2 (Phlpp2) mRNA m6A modification, thus alleviating cardiac dysfunction in myocardial I/R remodeling [59]. Moreover, exercise increases the expression of angiogenesis-related molecules and improves cardiac function in the myocardium of aged rats [60]. In addition, exercise can also increase sarcoplasmic/endoplasmic reticulum calcium ATPase 2a (SERCA2a) activity and myofilament responsiveness to calcium (${\mathrm{Ca}}^{2+}$) and improve the cellular ${\mathrm{Ca}}^{2+}$ influx mechanism, attenuating cardiac functional impairment in failing hearts [61].

In summary, exercise exhibits a range of potential mechanisms for protecting against HF, such as reducing apoptosis, modulating signaling pathways, influencing the release of peptides, regulating gene expression, promoting angiogenesis, and optimizing intracellular ${\mathrm{Ca}}^{2+}$ flow. Based on different aetiologies of HF, exercise may exert protection through diverse mechanisms. However, further research is necessary to fully understand and unravel the intricacies of these mechanisms.

## 4. Conclusions

Heart failure remains a significant global cause of mortality, highlighting the need for effective interventions. Guidelines currently recommend exercise training as an essential component of therapy for heart failure patients. This review emphasizes that exercise plays a crucial role in protecting against heart failure through mechanisms such as alleviating cardiac fibrosis, reducing inflammation and regulating mitochondrial metabolism. As such, exercise represents a potential target for interventions aimed at preventing and treating heart failure. However, the precise role and specific underlying mechanisms of exercise in heart failure are still not fully understood. Therefore, further exploration of exercise in the context of heart failure is necessary to gain new insights and provide more effective strategies for the prevention and treatment of this clinical condition.
